# Synthesis of Two-Dimensional Zeolite Nanosheets Applied to the Catalytic Cracking of a Waste Cooking Oil Model Compound to Produce Light Olefins

**DOI:** 10.1021/acsomega.3c08748

**Published:** 2024-04-02

**Authors:** Wenbo Luo, Haoyu Liu, Hong Yuan, Hao Liu

**Affiliations:** † School of Chemistry and Chemical Engineering, North Minzu University, Yinchuan 750021, China; ‡ State Key Laboratory of National Ethnic Affairs Commission Chemical Technology, North Minzu University, Yinchuan 750021, China; § Ningxia Key Laboratory of Solar Chemical Conversion Technology, North Minzu University, Yinchuan 750021, China

## Abstract

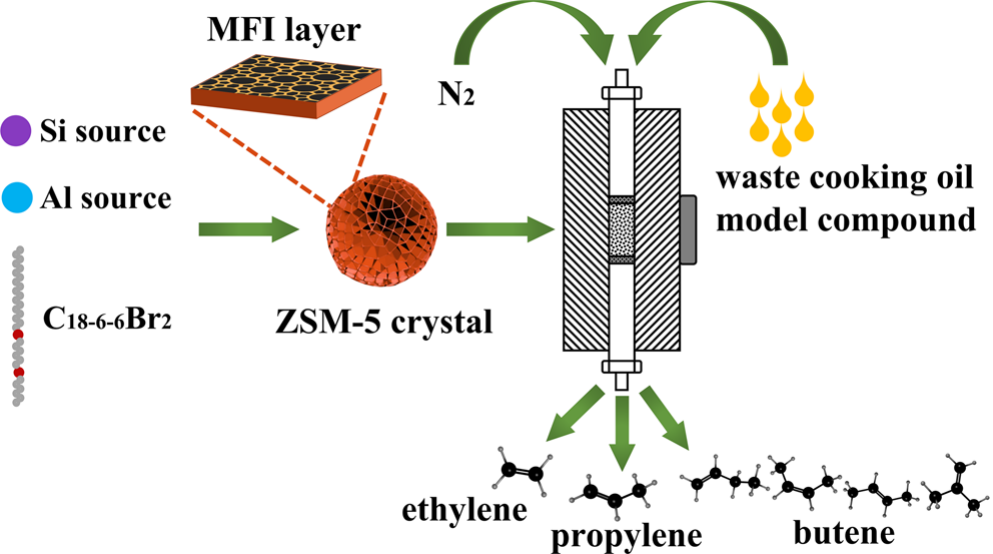

Hierarchical zeolites can provide multidimensional spatial networks and, therefore, have significant potential as catalysts for the cracking of biomass to generate light olefins. The present work synthesized the diquaternary ammonium-type surfactant [C_18_H_37_–N^+^(CH_3_)_2_–(CH_2_)_6_–N^+^(CH_3_)_2_–C_6_H_13_]Br_2_, incorporating hydrophobic 18-carbon alkyl groups for usage as a structure-directing agent. This compound was subsequently used to prepare nanosheets of a hierarchical ZSM-5 two-dimensional zeolite (HNZSM-5) through a one-pot hydrothermal method. The crystal phase, morphology, and hierarchical structure of the HNZSM-5 were analyzed using various techniques, including X-ray diffraction, electron microscopy, and N_2_ adsorption/desorption. When applied to the catalytic cracking of a waste cooking oil model compound, the HNZSM-5 exhibited superior activity and stability compared with a conventional ZSM-5. This performance was attributed to the more accessible acid sites and unique lamellar structure of the former material. The HNZSM-5 also outlasted the conventional zeolite, showing deactivation after 45 h of reaction compared with 20 h, indicating exceptional stability and excellent resistance to coking.

## 1 Introduction

Light olefins (ethylene, propylene, and butene) are important raw materials in the petrochemical industry and are widely used in the manufacture of polymers, solvents, additives, adhesives, and other products.^1−3^ These compounds are typically produced via the steam or catalytic cracking of naphtha, light diesel, and other petroleum products. Because catalytic cracking allows significantly reduced temperatures and the product distribution is readily tuned, this process has been widely studied and commonly employed in recent years.^4^ In addition, the catalytic cracking of biomass to produce light olefins has gradually attracted the attention of researchers.

The use of biomass has the advantage of promoting net zero CO_2_ emissions; therefore, it is expected to have a reduced impact in terms of the greenhouse effect.^5^ Hence, there has been much research in this field. Zhang et al.^6^ determined that the addition of CaO and Al_2_O_3_ during biomass pyrolysis promoted the conversion of oxygen-containing functional groups to generate olefins and improved the selectivity for light olefins. Wang et al.^7^ employed cellulose, hemicellulose, and lignin as raw materials to produce light olefins based on catalytic cracking, and the selectivity of ethylene in the C_1_–C_4_ products was around 50%. Waste cooking oil (WCO) contains primarily fatty acid triglycerides and free fatty acids. In particular, WCO has straight carbon chain structures similar to naphtha; therefore, it may serve as an ideal raw material for the preparation of light olefins.

The unique structures of ZSM-5 zeolites, which incorporate micropores with an average size of approximately 0.55 nm, provide suitable shape selectivity for light olefins. In addition, the high Bronsted acid densities of these materials can promote the cracking of oxygen-containing intermediates to generate light olefins. Consequently, much prior research has examined the use of these zeolites to catalytically crack biomass to produce light olefins. As an example, Gong et al.^8^ applied a La/HZSM-5 zeolite to the catalytic cracking of bio-oil to synthesize light olefins and found that the selectivity for and yield of light olefins could be significantly improved by adjusting the acid site density and distribution of this material. Li et al.^9^ used a La_2_O_3_-modified ZSM-5 to catalyze the cracking of oleic acid, methyl laurate, and WCO and obtained a product mixture comprising 10.7–15.4% ethylene, 13.6–17.1% propylene, and 5.3–6.3% butene.

However, Zhang et al.^10^ found that the catalytic cracking of biomass with ZSM-5 allowed macromolecular intermediates to collect on the surfaces of the zeolite where these compounds were converted into coke, leading to premature inactivation. In recent years, there have been many studies indicating that the microporous structure of ZSM-5 is not conducive to the diffusion of macromolecular substances and hence the catalytic activity of this material can be degraded.^11−13^ For this reason, researchers have worked hard to develop ZSM-5 zeolites having hierarchical pore structures that enhance the diffusion of molecules while retaining the shape selectivity of micropores.^14^ Vu et al.^15^ used a hierarchical ZSM-5 zeolite having a high proportion of mesopores to catalyze the cracking reactions of triolein and WCO. This prior work established that the introduction of mesopores improved the utilization of acid sites in the zeolite pores and that the raw material conversion rate and light olefin selectivity were better than those achieved with standard ZSM-5. Choi et al.^16^ successfully prepared MFI zeolite nanosheets having a hierarchical porous structure and a single unit cell thickness using a diquaternary ammonium-type surfactant [C_22_H_45_–N^+^(CH_3_)_2_–(CH_2_)_6_–N^+^(CH_3_)_2_–C_6_H_13_]Br_2_ (denoted as C_22–6–6_Br_2_) containing a long-chain alkyl group (C_22_) and two quaternary ammonium groups. Multilamellar stacking of these MFI nanosheets during three-dimensional intergrowth created numerous exposed active sites and produced many surface defects that improved the catalytic performance of the material. At the same time, the minimal thickness of the two-dimensional nanosheets in the *b*-axis dimension (only 2 nm) reduced the diffusion path compared with that of a conventional ZSM-5 zeolite, inhibiting coke deposition. Xiao et al.^17^ found that nanosheet ZSM-5 zeolites exhibited high activity and good anticoking stability during the catalytic cracking of *n*-heptane. These properties were attributed to the hierarchical porosity of this material along with the accessibility of acid sites and suitable diffusion properties of macromolecular substances within the zeolite. However, the use of the C_22–6–6_Br_2_ surfactant is expensive because of the cost of raw materials having C_22_ chains. In contrast, surfactants with C_18_ chains are relatively inexpensive. Even so, to date, research concerning the synthesis of zeolite nanosheets having hierarchical pore structures using C_18–6–6_Br_2_ as a structure-directing agent (SDA) has been uncommon.

In the present work, the diquaternary ammonium-type surfactant C_18–6–6_Br_2_ was synthesized and subsequently employed as an SDA to produce nanosheets of hierarchical ZSM-5 2D zeolite (HNZSM-5). The formation of this material was assessed using characterization techniques such as X-ray diffraction (XRD), field emission scanning electron microscopy (SEM), and transmission electron microscopy (TEM). The performance of the HNZSM-5 as a catalyst during the cracking of a waste cooking oil model compound (WCOMC) to produce light olefins was also investigated.

## 2 Experimental Section

### 2.1 Materials

The 1-bromooctadecane (C_18_H_37_Br, 97%), *N*,*N*,*N*,*N*-tetramethyl-1,6-hexanediamine (C_10_H_24_N_2_, 99%), 1-bromohexane (C_6_H_13_Br, 99%), sodium hydroxide (NaOH, 96%), aluminum sulfate octadecahydrate (Al_2_(SO_4_)_3_·18H_2_O, 99%), aluminum nitrate nonahydrate (Al(NO_3_)_3_·9H_2_O, 99%), tetraethyl orthosilicate (TEOS, 98%), soybean oil, and oleic acid used in this work were obtained from the Shanghai Aladdin Biochemical Technology Co., Ltd. (Shanghai, China). Tetrapropylammonium hydroxide (TPAOH, 25 wt % in water) was purchased from the Shanghai Macklin Biochemical Technology Co., Ltd. (Shanghai, China). Acetone (C_3_H_6_O, 99%), diethyl ether (C_4_H_10_O, 99%), sulfuric acid (H_2_SO_4_, 98%), and acetonitrile (C_2_H_3_N, 99%) were obtained from the Sinopharm Chemical Reagent Co., Ltd., China. The WCOMC was produced by heating 90 g of soybean oil at 150 °C for 5 h, then adding 10 g of oleic acid to the mixture, and stirring thoroughly.

### 2.2 Synthesis of HNZSM-5

The C_18–6–6_Br_2_ was synthesized using a method previously reported by Na et al.^18^ In this process, 16.67 g of C_18_H_37_Br and 43.08 g of C_10_H_24_N_2_ were combined in 200 mL of acetone followed by stirring at 60 °C for 16 h. After cooling to room temperature, the product was filtered. The C_18–6–0_Br obtained from this step was washed with ether three times and then dried in a vacuum drying oven at 50 °C under 0.014 MPa for 12 h. Subsequently, 15.18 g of C_18–6–0_Br and 9.91 g of C_6_H_13_Br were dissolved in 150 mL of acetonitrile followed by stirring and refluxing at 70 °C for 12 h. After cooling to room temperature, the product was filtered and then washed with ether three times. Finally, the material was dried at 50 °C under a pressure of 0.014 MPa for 12 h to obtain the C_18–6–6_Br_2_ (Figure S1).

The HNZSM-5 was synthesized according to a method reported by Choi et al.^16^ using C_18–6–6_ Br_2_ as the SDA. In this process, 1.15 g of NaOH was dissolved in 24.56 g of deionized water, after which 3.22 g of C_18–6–6_Br_2_ was added followed by stirring at room temperature for 0.5 h to obtain solution A. Simultaneously, 0.32 g of Al_2_(SO_4_)_3_·18H_2_O and 0.85 g of H_2_SO_4_ were dissolved in 10 g of deionized water to obtain solution B, after which solution B was added dropwise to solution A with stirring. The resulting mixture was stirred at 60 °C for an additional 1 h. After the solution was cooled to room temperature, 10.00 g of TEOS was added, and the mixture was stirred for a further 1 h at 60 °C. The resulting gel, having the molar ratios 30Na_2_O:1Al_2_O_3_:100SiO_2_:10C_18–6–6_Br_2_:18H_2_SO_4_:4000H_2_O, was crystallized by heating at 150 °C for 120 h, after which the solid product was washed several times with deionized water, dried at 120 °C for 12 h, and finally calcined in the air at 550 °C for 6 h. This gave the material referred to herein as HNZSM-5 (120 h). Additional specimens were produced by varying the crystallization time and are denoted herein as HNZSM-5 (24 h), HNZSM-5 (48 h), HNZSM-5 (72 h), and HNZSM-5 (96 h), where the value in brackets is the crystallization time.

### 2.3 Synthesis of Conventional ZSM-5 Zeolite

In this synthesis, 0.19 g of NaOH was dissolved in a solution comprising 9.49 g of TPAOH in 33.61 g of water by stirring at room temperature for 30 min. A 0.35 g quantity of Al(NO_3_)_3_·9H_2_O was then added to the mixture followed by stirring at 60 °C for 1 h. The solution was cooled to room temperature, and 9.72 g of TEOS was added followed by stirring at 60 °C for 1 h. The resulting gel, having the molar ratios 5Na_2_O:1Al_2_O_3_:100SiO_2_:25TPAOH:4000H_2_O, was transferred to an autoclave and crystallized by heating at 180 °C for 48 h, after which the solid product was repeatedly washed with deionized water and dried at 120 °C for 12 h. The dried product was calcined at 550 °C in the air for 6 h to obtain a conventional ZSM-5 zeolite (denoted herein as CZSM-5).

### 2.4 Characterization of Catalysts

Powder XRD patterns were acquired using a SmartLab diffractometer (Rigaku, Japan) operated at 40 kV and 30 mA with Cu Kα radiation. The scan speed was 4°/min, the step size was 0.02°, and the scanning range was 5–50°. The relative crystallinity of each specimen was calculated from the intensities of diffraction peaks in the 2θ range of 22–25° using the equation: crystallinity (%) = peak area of sample/peak area of standard × 100%. The original samples were used as standards in this process. Fourier transform infrared (FTIR) spectra were obtained using an FTIR-650 spectrometer (Guangdong, China) over the range of 4000–400 cm^–1^. SEM images of the powder samples were obtained with an EVO18 instrument (ZEISS, Germany) operating at 2–5 kV. TEM images were acquired using a JEM-F200 instrument (JEOL, Japan) at an accelerating voltage of 200 kV. Nitrogen (N_2_) adsorption–desorption isotherms were generated at −196 °C using an ASAP 2020 apparatus (Micromeritics). Prior to each analysis, the specimen was degassed by heating at 300 °C under vacuum for 6 h. Surface areas were calculated using the Brunauer–Emmett–Teller (BET) equation, and pore sizes were obtained from the maxima of the pore size distribution curves using the Barrett–Joyner–Halenda (BJH) method based on the adsorption branch of each isotherm. NH_3_ temperature-programmed desorption (NH_3_-TPD) profiles were produced using an AutoChem II 2920 chemisorption apparatus (Micromeritics). In each trial, a sample of approximately 60 mg was pretreated by heating at 500 °C with a 30 mL/min flow of Ar for 30 min and then cooled to 50 °C. The specimen was subsequently allowed to adsorb ammonia under a 30 mL/min flow of a 10% NH_3_/90% He mixture for 90 min. After switching to a 30 mL/min He purge for 60 min, the sample temperature was raised to 600 °C at 10 °C/min and desorbed NH_3_ was monitored using a thermal conductivity detector. Thermogravimetric analysis (TGA) was performed using an STA 200 apparatus (Hitachi, Japan) based on heating from 30 to 800 °C under an oxygen/helium mixture (10% oxygen by volume) flowing at 80 mL/min with a heating rate of 10 °C/min. The mass loss between 200 and 800 °C was used to calculate the amount of coke in each zeolite.

### 2.5 Catalytic Performance

**Figure 1:**
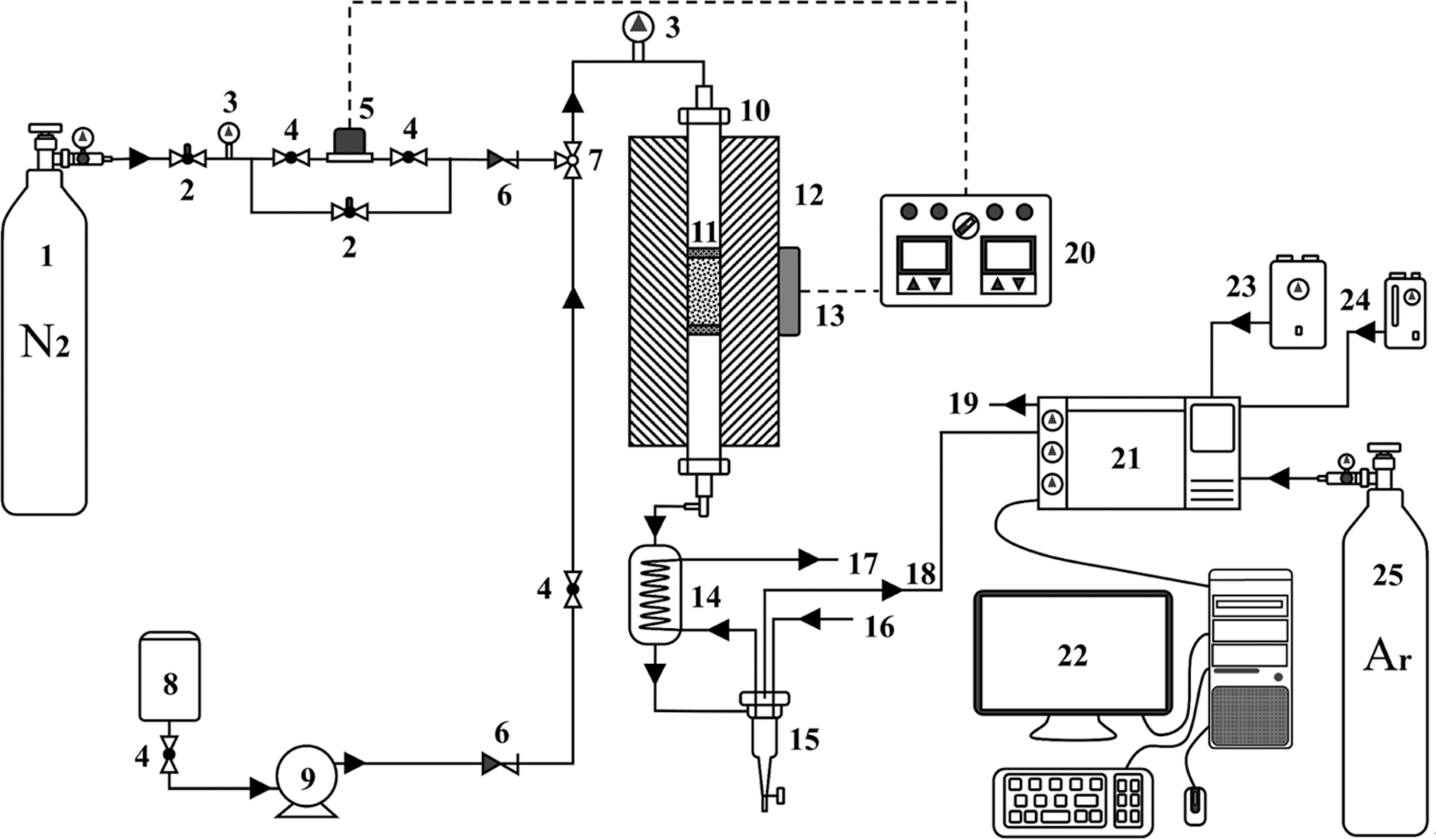
A diagram of the cracking reaction apparatus. Legend: 1. nitrogen cylinder, 2. control valve, 3. pressure indicator, 4. globe valve, 5. mass flowmeter, 6. check valve, 7. three-way valve, 8. feed storage tank, 9. metering pump, 10. tubular reactor, 11. catalyst bed, 12. heating furnace, 13. furnace thermocouple, 14. water-cooled condenser, 15. gas–liquid separator, 16. cooling water supply, 17. cooling water return, 18. gas outflow, 19. vent, 20. control panel, 21. gas chromatograph, 22. computer, 23. air generator, 24. hydrogen generator, 25. argon cylinder.

The WCOMC cracking reaction was carried out in a continuous-flow, fixed-bed stainless-steel tubular reactor (560 mm in length with a 12 mm inner diameter) under atmospheric pressure. A diagram of this apparatus is presented in Figure 1. Prior to the start of each catalytic reaction, 1 g of the catalyst was packed into a stainless-steel tubular reactor and the space above and below the catalyst bed was filled with quartz wool. The reactor was then heated to 300 °C, and the material was pretreated in a 30 mL/min flow of N_2_ for 2 h to desorb any impurities. Following this, the reactor was heated to a target temperature of 450, 500, 550, or 600 °C under 30 mL/min flow of N_2_. When the system stabilized, 0.04 mL/min flow of WCOMC was supplied to the catalyst bed via a 5963 Pump Optos 1LM metering pump (Eldex). The outlet stream was cooled in a gas–liquid separator so that the gaseous and liquid products were separately captured. These compounds were then analyzed using an online gas chromatograph (GC-7920, Huifen, China) equipped with a flame ionization detector and an HP-Al/KCl capillary column (30 m, 0.53 mm, 15 μm). Herein, the reaction products are categorized as dry gases (such as CH_4_, and C_2_H_6_), light olefins (such as C_2_H_4_, C_3_H_6_, and C_4_H_8_), and paraffins (such as C_2_H_6_, C_3_H_8_, and C_4_H_10_). The yields of and selectivities for these various products were calculated using the equations
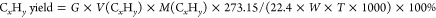
1where
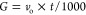
2

and
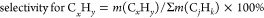
3Here, C_*x*_H_*y*_ represents the reaction product, *G* (L) is the gas production, *V* (C_*x*_H_*y*_, %) is the volume percentage of C_*x*_H_*y*_ species in the gas product, *M* (C_*x*_H_*y*_, g/mol) is the relative atomic mass of the C_*x*_H_*y*_ species, *W* (kg) is the quality of the raw material input, *T* represents the temperature, which is 303.15 K, v_o_ (mL/min) is the flow rate of gas products, *t* (min) is the reaction time, *m* (C_*x*_H_*y*_, %) is the mass fraction of the product C_*x*_H_*y*_, and ∑*m* (C*_j_*H*_k_*, %) is the sum of the mass fraction of products.

## 3 Results and Discussion

### 3.1 HNZSM-5 Obtained with Varying Crystallization Times

**Figure 2:**
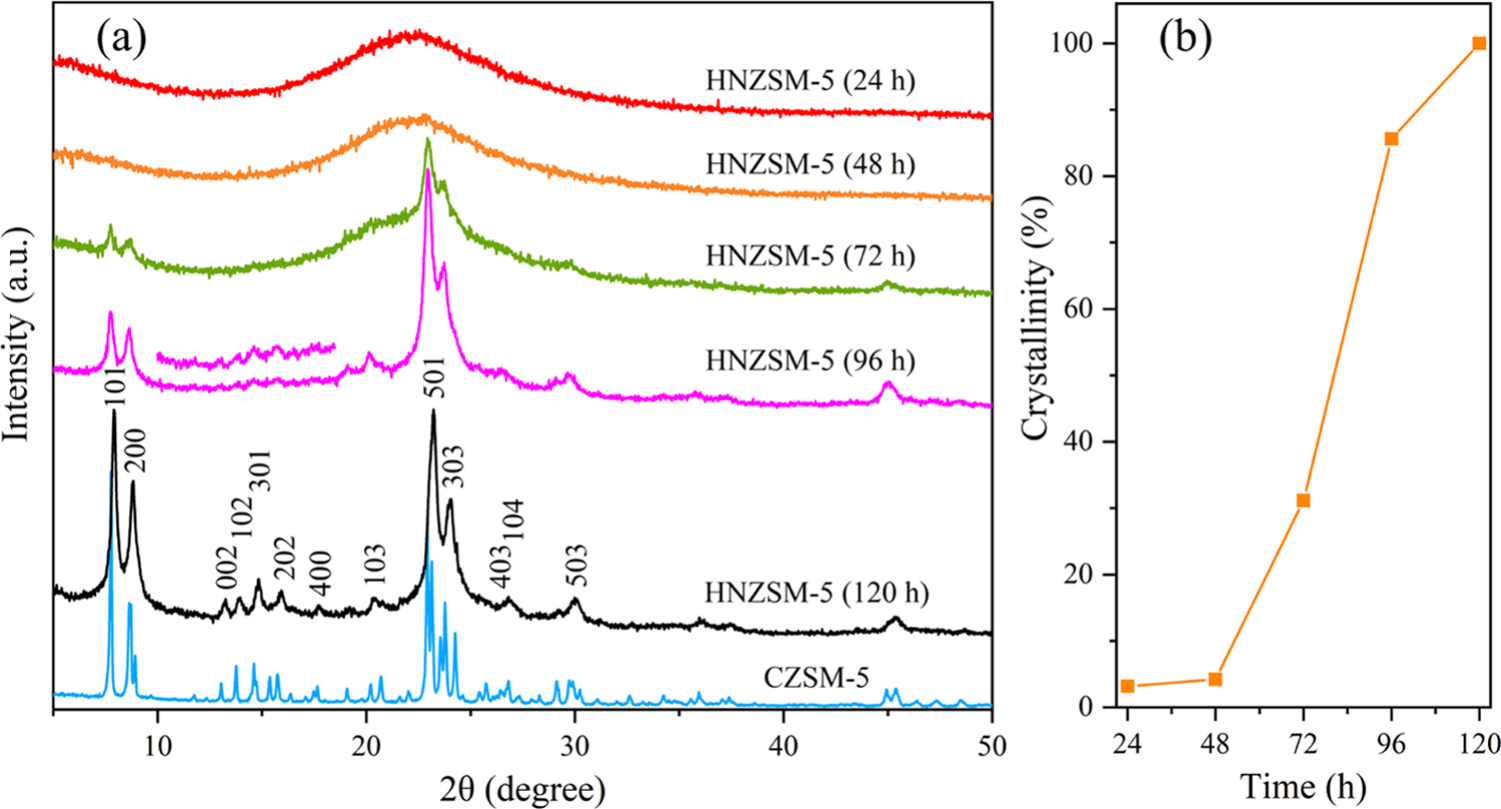
(a) XRD patterns of HNZSM-5 specimens produced using different crystallization times and of a CZSM-5 sample. (b) Extent of HNZSM-5 crystallinity as a function of the growth time.

The XRD patterns of the HNZSM-5 specimens obtained with different crystallization times are presented in Figure 2a, while the crystallinity of these materials is plotted as a function of crystallization time in Figure 2b. Crystallization times of 24 and 48 h gave flattened diffraction peaks in the range of 2θ = 15–30°, indicating that the tetraethyl silicate was hydrolyzed to form amorphous nanoparticles. As the size of these particles increased, the particles aggregated and gradually nucleated.^19,20^ The very low crystallinity of these specimens (<10%) shown in Figure 2b suggests that a large proportion of intermediate products was present as an amorphous phase after 48 h of crystallization. When the crystallization time was increased to 72 h, the crystallinity of the material was increased and peaks appeared at 7.9, 8.9, 23.1, and 24.0°. From the perspective of the charge effect, organic amine cations easily form H-bond complexes with the Si–OH terminal groups of the silicate anion.^21^ Therefore, the quaternary ammonium groups in C_18–6–6_Br_2_ acted as structure-directing species, enabling the amorphous gel to form MFI topological features, thereby promoting the crystallization of zeolite framework structures. Following 96 h of crystallization, the SDA caused the crystallinity to undergo a sudden rapid increase, and the characteristic peaks associated with an MFI structure in the XRD pattern became sharper and more intense. These data demonstrate that a highly crystalline MFI structure emerged. In addition, less intense peaks were obtained in the range of 2θ = 10–20°, and peaks related to (103) and (503) crystal plane diffractions appeared at 20.23 and 29.92°. These results established that the hydrophobic 18-carbon chains limited growth along the *b*-axis, resulting in wide *a*–*c* planes and a thin-layer structure with a short *b*-axis. In the case that the crystallization was extended to 120 h, the peaks generated by the HNZSM-5 were broadened, and only the (*h*0*l*) crystal plane reflections remained sufficiently sharp to allow indexing. In addition, the peaks associated with the *b*-axis crystal plane disappeared. The sample was evidently highly crystalline so that peaks related to a lamellar structure were obtained, confirming that a ZSM-5 zeolite having a short *b*-axis had been synthesized.

**Figure 3:**
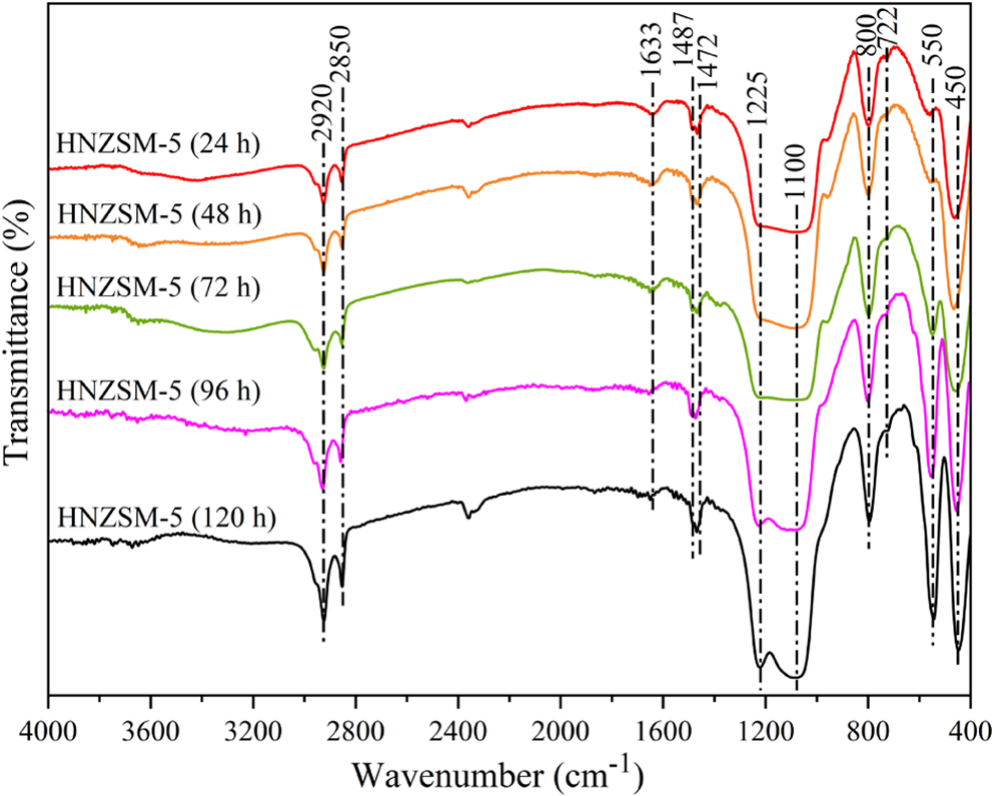
FTIR spectra of HNZSM-5 specimens (before calcination) produced by using various crystallization times.

**Figure 4:**
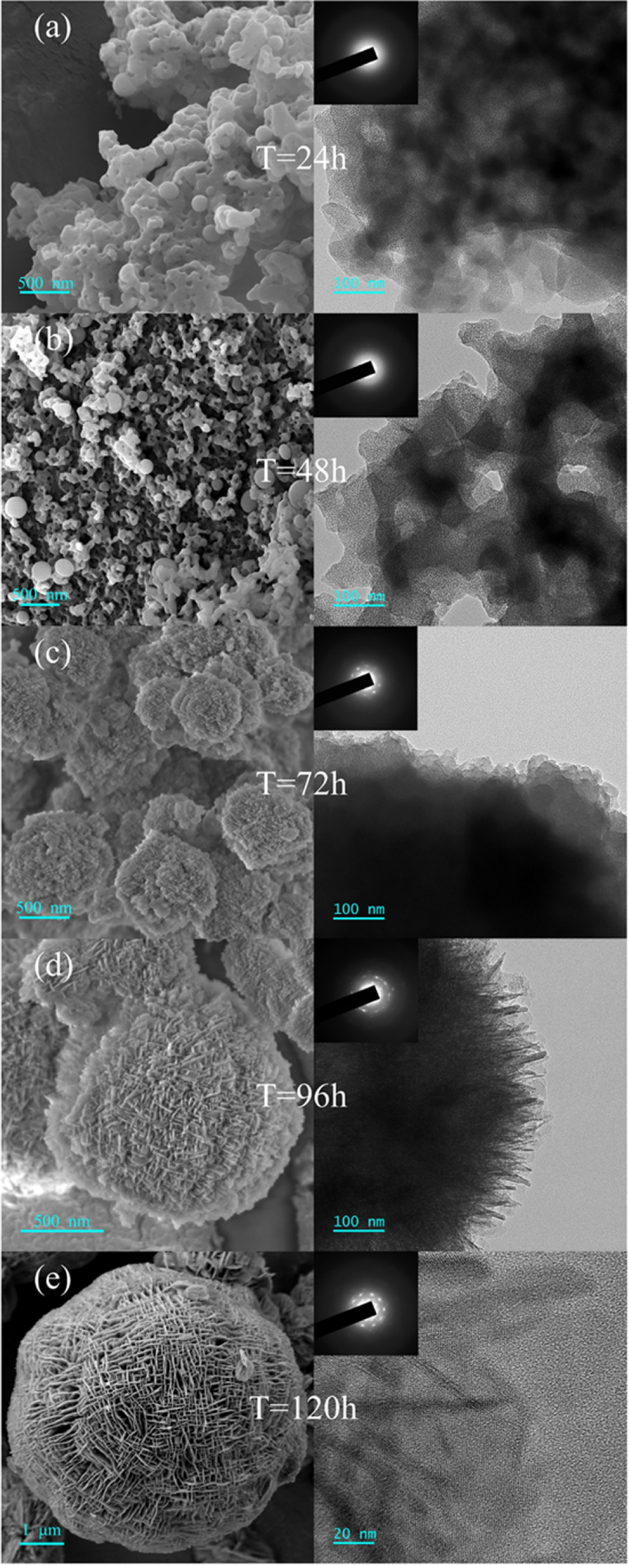
SEM (left) and TEM (right) images of HNZSM-5 specimens produced by using different crystallization times.

The FTIR spectra acquired from HNZSM-5 specimens prior to calcination as generated by using different crystallization times are shown in Figure 3. Here, the peaks related to the zeolite framework appear in the range 400–1300 cm^–1^. Specifically, the peak at 450 cm^–1^ corresponds to the bending vibration of T–O bonds (T = Si, Al) in TO_4_ tetrahedra. In addition, the peaks at 550 and 1225 cm^–1^ are respectively attributed to the asymmetric stretching of Si–O–T groups in five-membered rings and the external asymmetric stretching of TO_4_ tetrahedra. The peaks at 800 and 1100 cm^–1^ correspond to the external symmetric stretching and internal asymmetric stretching of siliceous materials, respectively.^22^ A comparison of the relative intensities of the peaks at 450 and 550 cm^–1^ in the spectra of the various specimens indicates that the crystallinity of HNZSM-5 gradually increased as the crystallization time was prolonged. The peaks at 550 and 1225 cm^–1^ were very weak after 24 and 48 h, demonstrating that a large portion of the material was in a gel or amorphous state, and the MFI topology was not fully formed. After 96 h, the intensity of the peak at 550 cm^–1^ was increased and an IR vibration band sensitive to the ZSM-5 framework appeared at 1225 cm^–1^. These data confirm that the MFI structure was produced, in agreement with the XRD results. Peaks characteristic of the SDA can be seen at 722, 1472, 1487, 2850, and 2920 cm^–1^. Among these, the peaks at 722 and 1472 cm^–1^ are ascribed to the bending vibration of −CH_2_– groups, whereas those at 2850 and 2920 cm^–1^ are related to the symmetric and asymmetric stretching vibrations of the same groups, and the peak at 1487 cm^–1^ is attributed to the bending vibration of −N^+^(CH_3_)_2_ groups.^23^ The formation of the MFI framework and the short *b*-axis lamellar structure of HNZSM-5 (Figure 4) resulted from the effect of the SDA during the hydrothermal synthesis. It is also apparent that the SDA remained in the HNZSM-5 and was not decomposed. Finally, the peak at 1633 cm^–1^ is attributed to water in the crystal lattice.

Both SEM and TEM images of HNZSM-5 specimens produced by using varying crystallization times are provided in Figure 4. After 24 h [Figure 4a], amorphous nanoparticles appeared on the gel surfaces. In addition, the TEM image of this specimen demonstrates that the large gel particles were composed of smaller particles with sizes on the order of 100 nm and that these nanoparticles were closely packed. The inset shows the selected area electron diffraction (SAED) image acquired from this sample, which confirms that the nanoparticle aggregates were amorphous. After crystallization for 48 h [Figure 4b], the SEM image shows that the smaller nanoparticles were transformed into larger, smooth spheres, while interconnected worm-like structures appear in the TEM image. After the crystallization time was extended to 72 h [Figure 4c], the amorphous gel was consumed, and some nanoparticles were evidently attached to the surfaces of the spheres. In addition, the crystallinity of the material was increased. The TEM image shows that the edges of the spheres were very rough, whereas diffraction spots can be seen in the SAED image. Figure S2a indicates a similar core–shell structure, suggesting that the smooth spheres acted as nuclei to produce the sheet-like crystallite phase of HNZSM-5 together with an MFI structure. After 96 h of crystallization [Figure 4d], both the SEM and TEM images demonstrate spheres composed of nanosheets. The hydrophilic groups in the present SDA are thought to have guided the formation of micropores, while the longer 18-carbon chains inhibited the growth of the framework along the *b*-axis. Nanoparticles previously attached to the surface eventually transitioned to flakes with well-developed *a*–*c* planes and short *b*-axis lengths. After crystallization for 120 h [Figure 4e], the SEM image demonstrates a nearly spherical three-dimensional structure together with lamellae having thicknesses of 10–20 nm with layered stacking. In addition, zeolite crystals were fully formed with a high degree of geometric regularity. Lattice fringes are clearly evident in the high-resolution TEM image, and the SAED inset in the figure exhibits obvious diffraction spots, indicating that the particle was highly crystalline. The HNZSM-5 nanosheets seem to have grown in the vertical direction and intersected with one another to form a hierarchical structure similar to that of the MFI zeolite reported by Choi et al.^16^ However, Choi et al. synthesized ultrathin zeolite nanosheets using a diquaternary ammonium-type surfactant based on hydrophobic 22-carbon chains. The shorter carbon chains in the present SDA appear to have reduced the interlayer spacing.^18^ As the SDA was removed by calcination, rapid condensation of the MFI layers resulted in deformation and stacking so that multilamellar MFI layers were formed [Figure S2b]. Adjacent MFI layers did not completely collapse during calcination because these layers supported one another, although the sheet thickness was increased [Figure S2c]. Interestingly, the thin-layer morphology of the HNZSM-5 resembled a house-of-cards structure built from repeatedly branching lamellae,^24^ while the hierarchical morphology was similar to that of a self-pillared pentasil zeolite.^25^

**Figure 5:**
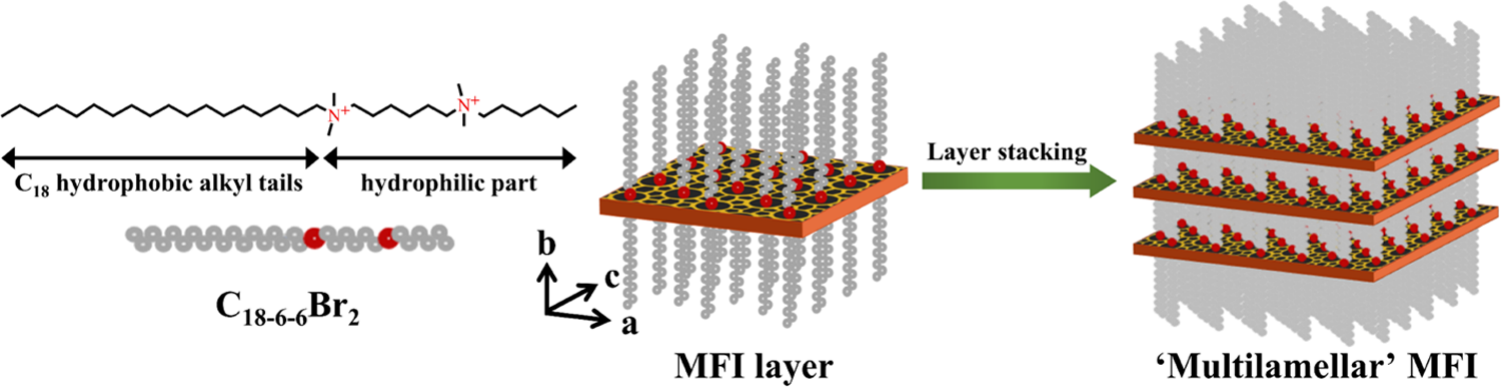
Diagrams showing the molecular structure of C_18–6–6_Br_2_ and the role of this compound in guiding the zeolite synthesis process.

As shown in Figure 5, C_18–6–6_Br_2_ was composed of a hydrophilic headgroup (two quaternary ammonium groups) and a hydrophobic long-chain alkyl group (C_18_). During the growth of the zeolite, the hydrophilic headgroup of the SDA guides the synthesis of the micropores, and the hydrophobic effect of the long-chain alkyl group inhibits the growth of zeolite in the *b*-axis direction. Therefore, a lamellar structure with a wide *a*–*c* plane and a short *b*-axis is formed. The lamellae stack with each other, and the interlayer pores provide mesopores. In this way, a hierarchical porous structure containing both micropores and mesopores is formed.

**Figure 6:**
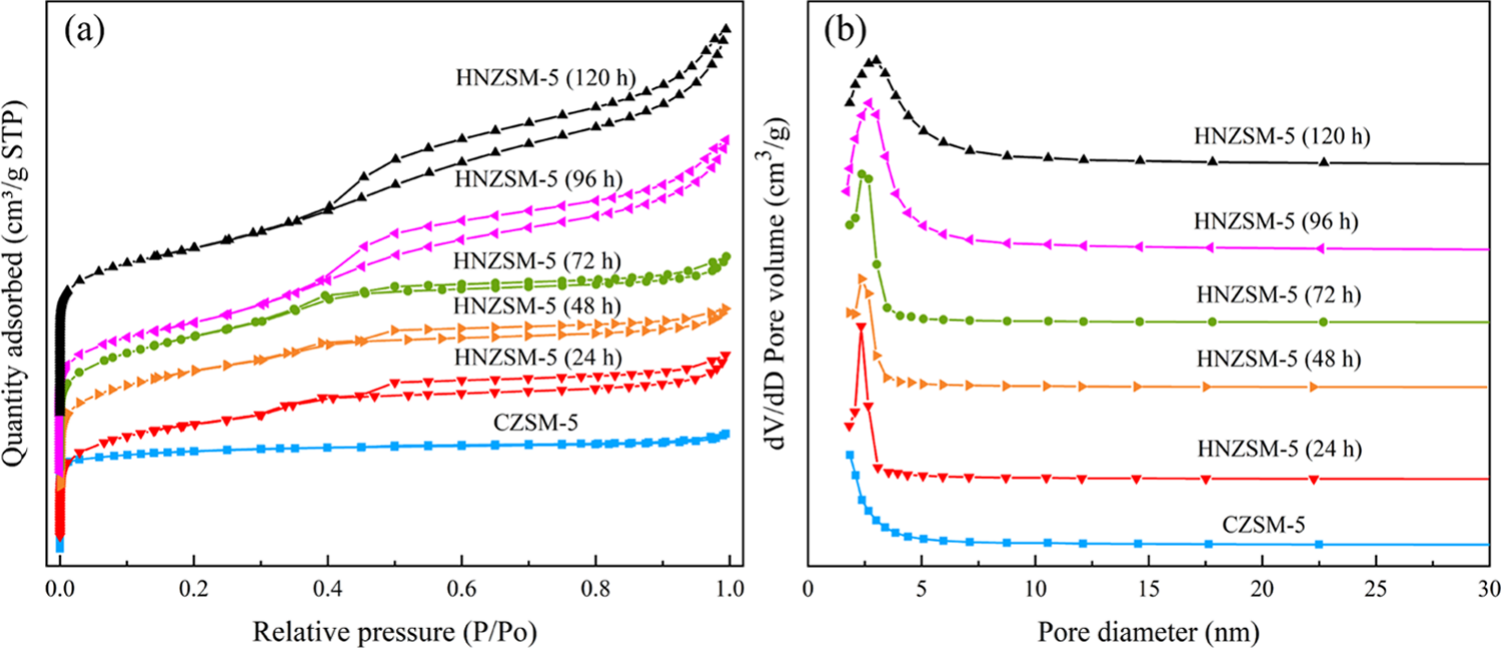
(a) N_2_ adsorption/desorption isotherms and (b) pore size distribution curves obtained from HNZSM-5 specimens produced using different crystallization times and from CZSM-5 samples.

The N_2_ adsorption/desorption isotherms and corresponding pore size distributions of HNZSM-5 specimens produced using different crystallization times and a CZSM-5 sample are provided in Figure 6. Each of the HNZSM-5 specimens generated a type-IV isotherm, indicating that both micropores and mesopores were present.^26^ After a crystallization time of 24 h, the isotherm exhibited a typical H_4_ hysteresis loop in the relative pressure (*P*/*P*_0_) range of 0.4–1.0, suggesting the presence of mesopores in the sample. Figure 4a shows that the surface of the gel was rough and that “wormholes” appeared after calcination, together with gaps between large particles that formed mesopores. After the crystallization time was extended to 48 h, a similar isotherm was obtained. The absorption at high *P*/*P*_0_ values is attributed to pores formed between the small amorphous gel particles. Compared with the HNZSM-5 (24 h), the pore volume of the HNZSM-5 (48 h) was not changed significantly, although the *S*_ext_ increased as the particle sizes became larger (Table 1). In the case of the HNZSM-5 (72 h), the hysteresis loop was significantly smaller as a result of the loss of the gel phase, and a type-I isotherm was obtained. This result implies the appearance of an MFI framework, which is consistent with the XRD and FTIR results. The hysteresis loop produced at high *P*/*P*_0_ values changed from type H_4_ to H_3_ after 96 h, establishing that mesopores appeared in the randomly stacked nanosheets.^27^ At this point, the *S*_BET_ of the material rapidly increased based on the increase in the micropore surface area associated with the highly crystalline MFI framework. In addition, the *V*_total_ and *D*_aver_ of the sample were increased to 0.57 cm^3^/g and 4.00 nm, respectively, indicating that the hydrophilic groups and 18-carbon groups, respectively, promoted the formation of micropores and mesopores.^28^ In contrast to the HNZSM-5 (120 h), the CZSM-5 provided a type-I isotherm characteristic of a microporous material.^29^ This was confirmed by the BJH pore size distribution for this specimen, [Figure 6b], in which no peak was present in the mesopore range. Figure 6b demonstrated that the pore size of the HNZSM-5 (120 h) sample was primarily within the range of 2–10 nm, meaning that mesopores were uniformly distributed. Hence, C_18–6–6_Br_2_ served as an SDA for the synthesis of hierarchically porous HNZSM-5 zeolites. These materials retained the original micropores of CZSM-5 but also allowed for more accurate control of the mesopore size and exhibited improved structural integrity.

**Table 1:** Textural Properties of HNZSM-5 Specimens Produced Using Different Crystallization Times and of a CZSM-5 Sample

	surface area (m^2^/g)			pore volume (cm^3^/g)			pore size (nm)
samples	*S*_BET_^a^	*S*_micro_^b^	*S*_ext_^c^	*V*_total_^d^	*V*_micro_^e^	*V*_meso_^f^	*D*_aver_^g^
CZSM-5	334.82	250.87	83.95	0.20	0.13	0.07	2.37
HNZSM-5 (24 h)	412.97	132.73	280.24	0.31	0.07	0.24	3.00
HNZSM-5 (48 h)	433.44	104.11	329.33	0.30	0.05	0.25	2.81
HNZSM-5 (72 h)	501.02	97.95	403.07	0.36	0.05	0.31	2.89
HNZSM-5 (96 h)	564.87	186.72	378.15	0.57	0.09	0.48	4.00
HNZSM-5 (120 h)	609.40	236.14	373.26	0.65	0.12	0.53	4.28

a *S*_BET_ determined by the BET method.

b Calculated using the *t*-plot method.

c *S*_ext_ = *S*_BET_ – *S*_micro_.

d Obtained from the amount adsorbed.

e Calculated using the *t*-plot method.

f *V*_meso_ = *V*_total_ – *V*_micro_.

g Average pore size from N_2_ adsorption isotherms.

**Figure 7:**
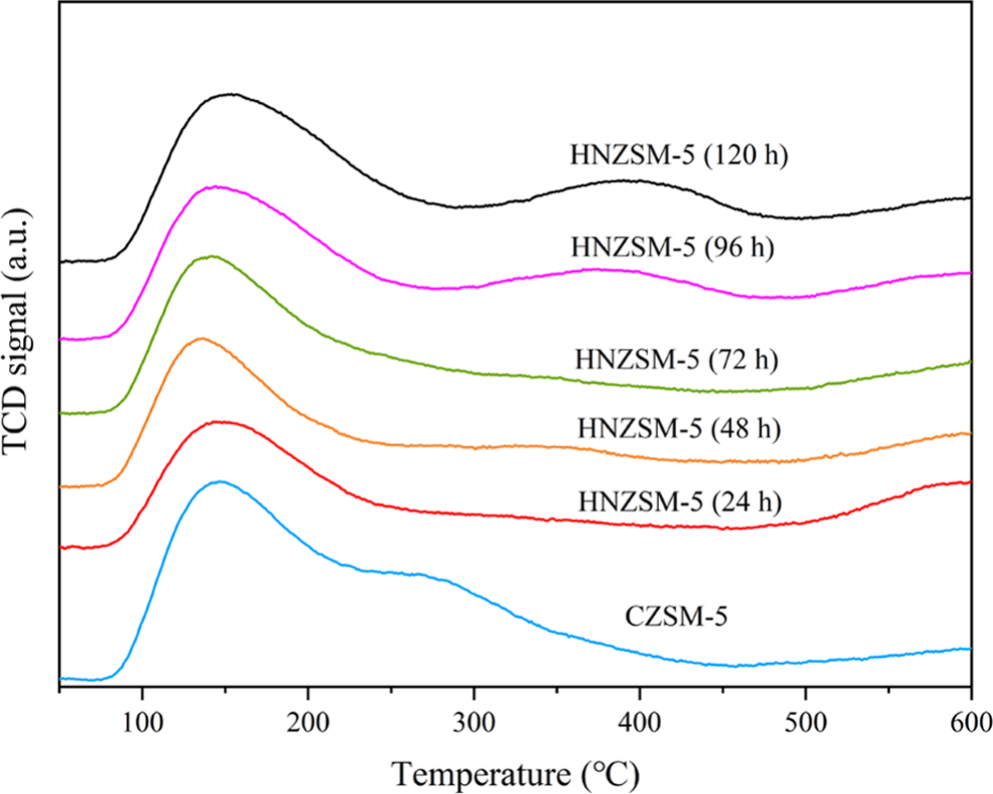
NH_3_-TPD curves of HNZSM-5 specimens produced by using different crystallization times and of a CZSM-5 sample.

The acidic properties of HNZSM-5 specimens made using different crystallization times and of a CZSM-5 sample were investigated by NH_3_-TPD, as summarized in Figure 7 and Table 2. Here, each plot shows two peaks in the ranges of 160–180 and 300–340 °C, corresponding to weak and strong acid sites, respectively.^30^ The low-temperature peak is usually ascribed to hydroxyl groups (−OH) bonded to defect sites, such as in the case of Si–OH and Al–OH groups, to produce weak Lewis acid sites. The high-temperature peak is assigned to Si–OH–Al groups that correspond to Bronsted or strong Lewis acid sites.^31,32^ Comparing the desorption curves of the HNZSM-5 samples produced using various crystallization times, it is apparent that prolonging the crystallization time increased the desorption signal intensity. This result confirms that the quantities of weak and strong acid sites were gradually increased. Compared with the HNZSM-5 (48 h), the amount of strong acid sites in the sample crystallized for 72 h was increased (Table 2). Hence, more Si–OH–Al groups were formed in the latter, meaning that an MFI framework was established. This finding is consistent with the XRD and FTIR results. After 96 h of crystallization, the NH_3_ desorption peak at the higher temperature became more intense and moved to an elevated temperature. Thus, a highly crystalline MFI framework with numerous strong acid sites was obtained. Table 2 shows that the number of acid sites associated with NH_3_ desorption increased significantly at this point. Hence, there were abundant intragranular mesopores in HNZSM-5 (96 h), resulting in more accessible acid sites. The total acid content of the CZSM-5 was higher than that of the HNZSM-5 (120 h), and the former contained many more strong acid sites while the latter had more weak acid sites. This difference can be ascribed to the formation of a lamellar structure with more defects.

**Table 2:** Acid Properties of the Samples

	acidity (mmol/g)		
samples	weak	strong	total
CZSM-5	0.61	0.45	1.06
HNZSM-5 (24 h)	0.41	0.17	0.58
HNZSM-5 (48 h)	0.45	0.18	0.63
HNZSM-5 (72 h)	0.45	0.22	0.67
HNZSM-5 (96 h)	0.58	0.35	0.93
HNZSM-5 (120 h)	0.66	0.36	1.02

### 3.2 Catalyst Performance

**Figure 8:**
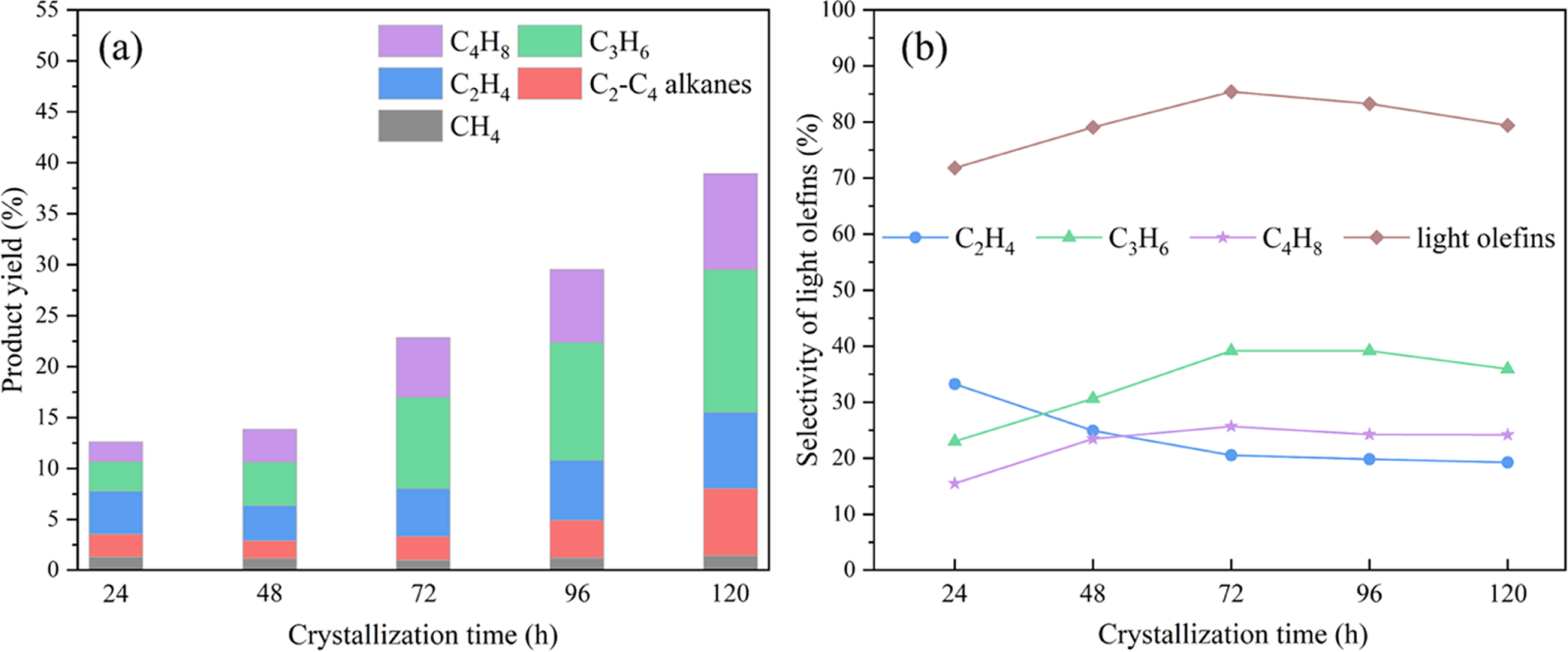
(a) Product yields and (b) light olefin selectivities obtained from the catalytic cracking of WCOMC with HNZSM-5 specimens produced using different crystallization times. Reaction conditions: temperature = 500 °C, time = 12 h, and WCOMC flow rate = 0.04 mL/min.

The performance of HNZSM-5 with different crystallization times in the catalytic cracking of WCOMC is summarized in Figure 8a. As the crystallization time of the catalyst was increased from 24 to 120 h, the yield of the obtained reaction products (dry gases, light olefins, and paraffins) gradually increased. When the HNZSM-5 (48 h) catalyst was used, the product yield was 13.9%. For this catalyst, a large number of amorphous intermediates remained and there were few strong acid sites, leading to low effectiveness in catalytic cracking. After 72 h of crystallization, the MFI skeleton structure with strong acid sites of the catalysts began to appear. The presence of strong acid sites promoted catalytic cracking of WCOMC, resulting in a considerable increase in the yield of the obtained product. The yield of the product obtained from the catalyst crystallized for 96 h further increased. Compared with HNZSM-5 (72 h), HNZSM-5 (96 h) had a higher *S*_BET_ (Table 1) and a greater number of strong acidic sites (Table 2), which contributed to the complete cracking of WCOMC. The highest yield was obtained from HNZSM-5 (120 h), which was 38.9%. The highly crystalline MFI framework structure provided many acidic sites required for WCOMC cracking. Moreover, the large mesoporous volume and *D*_aver_ value (Table 1) contributed to enhanced mass transfer of the macromolecular reactants. These effects promoted contact between the reactants and acidic sites, thereby increasing the obtained yield.

Figure 8a,b show the yield and selectivity of light olefins obtained from HNZSM-5 formed at different crystallization times. The yields of light olefin obtained from the HNZSM-5 (24 h) and HNZSM-5 (48 h) catalysts were 9.1 and 11.0%, respectively. Owing to the poor effectiveness of amorphous particles in cracking WCOMC, these catalysts gave low yields of light olefins. After 72 h of crystallization, the appearance of the MFI skeleton structure in the catalyst improved the yield of light olefins. For example, HNZSM-5 (72 h) gave the highest selectivity for light olefins. Although the selectivity for light olefins of HNZSM-5 (96 h) and HNZSM-5 (120 h) was lower than that of HNZSM-5 (72 h), HNZSM-5 (120 h) still achieved a maximum light olefin yield of 30.9% [Figures 8a]. The abundance of acidic sites and high *S*_BET_ of HNZSM-5 (120 h) promoted the cracking of the long carbon chain structure of WCOMC into small molecular products while also promoting the production of light olefins.

**Figure 9:**
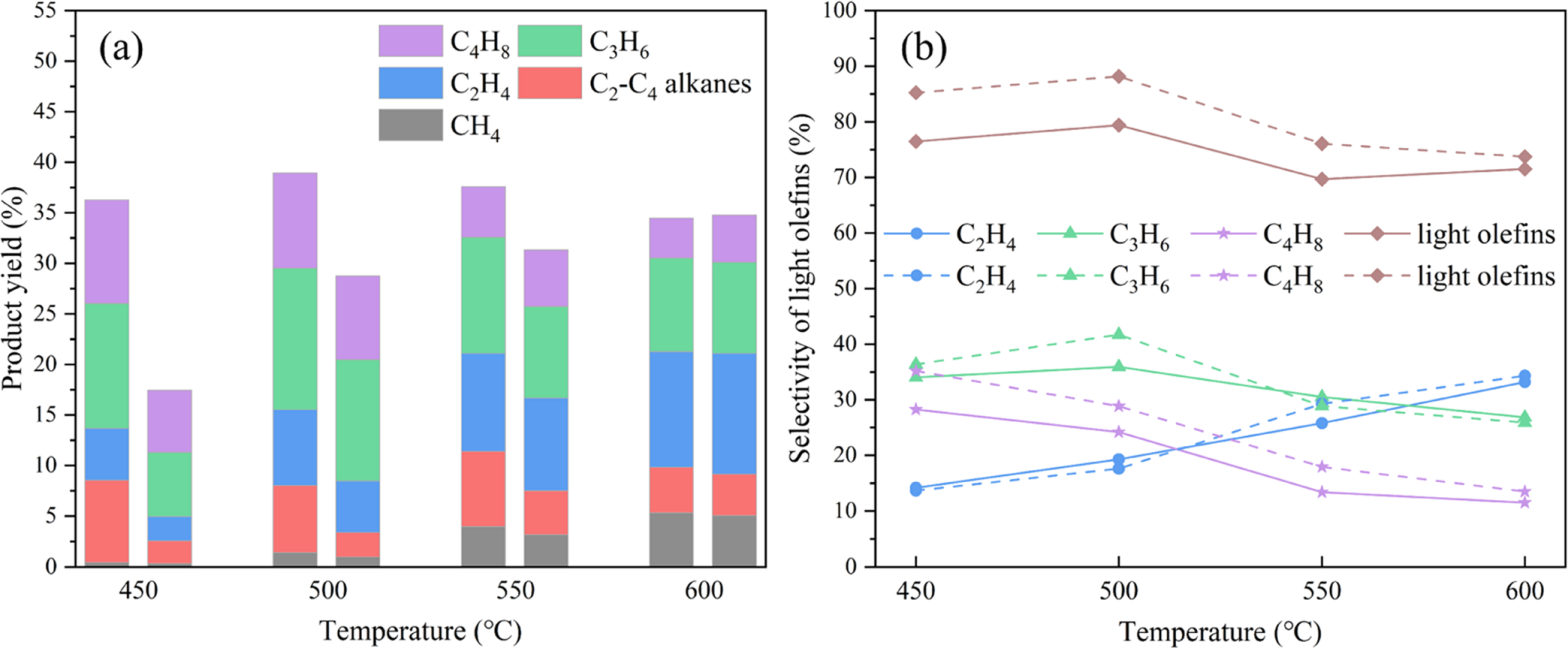
(a) Product yields (left: HNZSM-5, right: CZSM-5) and (b) light olefin selectivities (solid line: HNZSM-5, dotted line: CZSM-5) obtained during the catalytic cracking of WCOMC by using HNZSM-5 and CZSM-5 at various temperatures. Reaction conditions: time = 12 h and WCOMC flow rate = 0.04 mL/min.

The performances of HNZSM-5 (120 h) and CZSM-5 during the catalytic cracking of WCOMC over the temperature range of 450–600 °C are summarized in Figure 9. As the reaction temperature was increased from 450 to 500 °C, the yield of reaction products (dry gases, light olefins, and paraffins) obtained from the HNZSM-5 increased from 36.3 to 38.9%. However, as the temperature was further increased, this yield began to show a downward trend, likely because side reactions such as aromatization and coke production from light olefins intensified. In the case of the CZSM-5, increasing the temperature from 450 to 600 °C gradually increased the yield. Below 600 °C, the HNZSM-5 outperformed the CZSM-5. This outcome was attributed to the cross-stacking of the HNZSM-5 flakes, which provided a larger BET surface area and abundant mesopores (Table 1). These factors likely improved the access of reactants to acidic sites, together with the mass transfer efficiency of macromolecular intermediates, resulting in high yields. In contrast, the CZSM-5 had a lower surface area and a high acid content (Tables 1 and 2) but with acidic sites primarily located in micropores.^33^ At 600 °C, the yield obtained from CZSM-5 was slightly higher than that produced by the HNZSM-5. Evidently, higher temperatures helped to break the WCOMC into low-molecular-weight hydrocarbons, thereby improving the utilization of acidic sites in the micropores of the CZSM-5. The reaction temperature also had an obvious effect on the product distribution. During the heating of the WCOMC, both thermal and catalytic cracking occurred simultaneously, and these processes were affected differently by temperature. Specifically, the former process, which involved radical chain reactions, was much more sensitive to temperature.^34^ As such, raising the temperature increased the yield of thermal cracking products such that the amount of methane increased monotonically with the temperature. Shi et al.^35^ used a fixed fluidized bed for fluid catalytic cracking of waste cooking oil on the LDO-75 catalyst. Their results showed that as the reaction temperature was increased from 400 to 550 °C, the C_1_–C_3_ components increased from 50.88 to 61.97%, whereas the C_4_–C_5_ components decreased from 38.38 to 26.11%. This behavior also indicates that high temperatures promote the cracking of raw materials to generate small molecular substances. Additionally, owing to the fact that waste cooking oil is mainly composed of unsaturated acids, the content of alkenes produced is much higher than that of alkanes.

Figure 9a exhibits that the total yield of light olefins provided by the HNZSM-5 catalyst first increased and then decreased with increases in temperature and that the maximum yield of light olefins, obtained at 500 °C, was 30.9%. Excessively high temperatures promoted secondary cracking of the light olefins and so decreased the yield of these products above 500 °C. Olefins were formed by further cracking of short-chain hydrocarbons,^36^ and a high temperature of 600 °C promoted the cracking of the WCOMC to produce more short-chain hydrocarbons. This effect improved the utilization of acidic sites in the micropores of CZSM-5, thereby increasing the yield of light olefins to 25.6%. This value that obtained from HNZSM-5 (24.7%) exceeded. However, a greater energy input would be required for this higher temperature, and the yield was much lower than that obtained from the HNZSM-5 at 500 °C. The HNZSM-5 had superior acid site accessibility and so provided a higher olefins yield through catalytic cracking with lower energy consumption compared with the CZSM-5. This outcome provides further evidence for the important role of acid sites in converting intermediates into lighter, more valuable hydrocarbons.^37^ To improve the yield of light olefins, hydrogenated vegetable oil (HVO) as a raw material has been studied. For example, Karaba et al.^38^ used a steam cracking process to crack HVO and found that at a reaction temperature of 800 °C, the obtained ethylene yield was in the range of 39.9–45.6 wt %, and the propylene yield was in the range of 18.7–19.2 wt %. Moreover, the yields of ethylene and propylene from HVO were significantly higher than those for atmospheric gas oil and hydrocracked vacuum distillate used as reference traditional feedstocks.

Figures 9a,b demonstrate that the reaction temperature affected both the product distribution and the selectivity for light olefins. As the reaction temperature was increased above 500 °C, the ethylene yield and the selectivity for this product for both catalysts gradually increased, reaching maximum values at 600 °C. Conversely, the yields and selectivities for propylene and butene gradually decreased. The main products of the oligomerization reaction of C_2_H_4_ at low temperatures were C_3_H_6_ and C_4_H_8_. As the temperature increased, the oligomerization reaction of C_2_H_4_ was greatly inhibited^39^ and the volume fraction of C_2_H_4_ increased significantly. Ding et al.^40^ studied the steam cracking of soybean oil and naphtha and found that the ethylene yield of soybean oil at 750 °C was 22.84%, which was higher than that of naphtha. When the temperature was above 775 °C, the ethylene yield of naphtha was higher than that of soybean oil. Therefore, the temperature is an important factor affecting the yield of ethylene. The selectivity of the CZSM-5 for light olefins was also generally higher than that of the HNZSM-5, possibly as a consequence of the shape selectivity of the micropores in the former material.^33^ Despite this, the HNZSM-5 still gave the highest yield of light olefins at 500 °C. Hence, the performance of these catalysts was determined by a balance between suitable acidity and good molecular diffusion properties, rather than selectivity. Therefore, the conditions employed during the cracking reaction and the physical and chemical properties of the zeolite catalyst (such as the acidity and pore structure) are important factors that can affect the production of light olefins.

**Figure 10:**
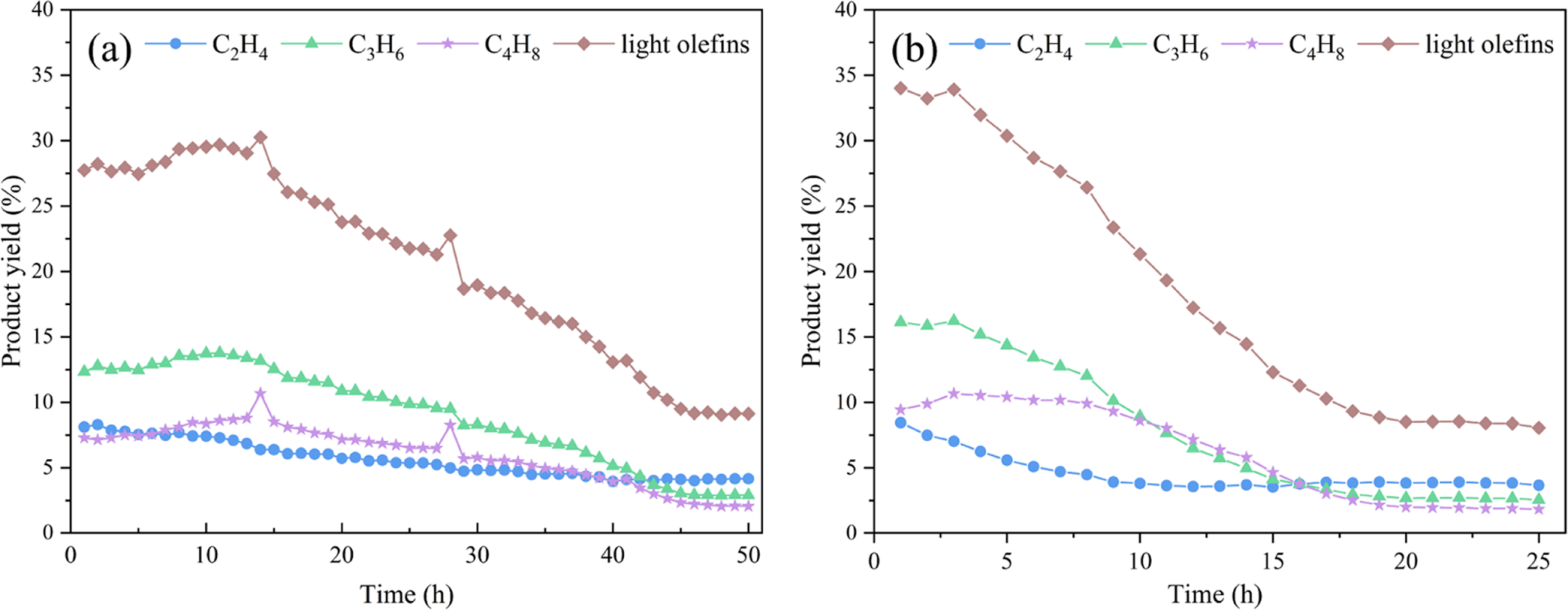
Product yields from the cracking of WCOMC using (a) HNZSM-5 and (b) CZSM-5 as functions of time. Reaction conditions: temperature = 500 °C, WCOMC flow rate = 0.04 mL/min.

**Figure 11:**
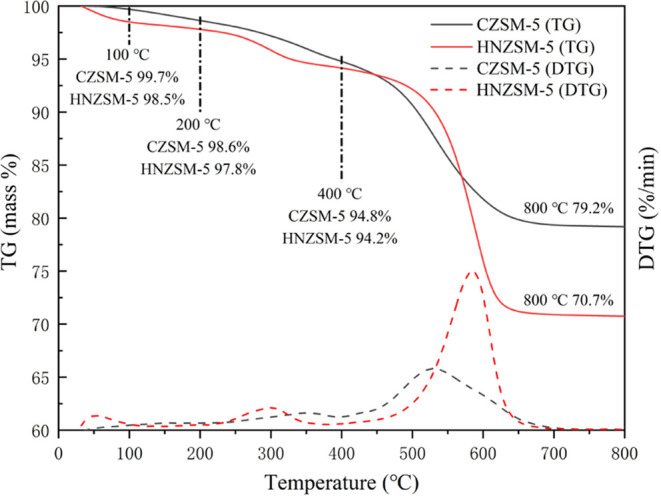
TG and DTG curves obtained from the spent HNZSM-5 and CZSM-5 catalysts.

Figure 10 plots the product distributions obtained over time using HNZSM-5 and CZSM-5 at 500 °C. In the case of HNZSM-5, the yield of light olefins was stable over the first 15 h, after which the yield began to decline. After 45 h, the yield of light olefins dropped to a minimum value and remained unchanged, indicating that the catalyst had been deactivated. In contrast, the yield of light olefins from the CZSM-5 decreased significantly beginning at 3 h, and the catalytic lifetime was only on the order of 20 h. The latter was significantly less stable during this reaction. These catalysts were primarily deactivated via the deposition of coke. The micropores in the CZSM-5 restricted diffusion, and the material had few surface acid sites. Hence, the access of macromolecular compounds to the active sites in the pores was limited. Coke was formed by polymerization, cyclization, and dehydrogenation reactions and eventually covered active sites on the catalyst while also blocking access to the pores, causing the catalyst to become less active.^41,42^ The mesopores in the HNZSM-5 served as storage sites for coke during the catalytic process, which prevented the clogging of micropores and improved the anticoking ability of the catalyst. The mesopores also significantly enhanced the diffusion performance of the material, thus reducing the probability of side reactions and further extending the life of the catalyst.^43,44^ The TGA results (Figure 11) for the two catalysts after 12 h of reaction under the same conditions show that HNZSM-5 contained more coke than the CZSM-5. Even so, the former maintained its catalytic activity during the subsequent operation to a greater extent. This finding demonstrates that the abundant external specific surface area of this specimen allowed the material to accommodate carbon deposits while leaving many active sites accessible for the reactant molecules.

### 3.3 TGA

Figure 11 presents the TG and DTG curves obtained from the HNZSM-5 and CZSM-5 after these materials were employed to promote the catalytic reaction at 500 °C. The mass loss from these specimens below 100 °C is attributed to the evaporation of physisorbed water from the zeolites, whereas that in the range from 100 to 200 °C resulted from the loss of intercrystalline water in the zeolite framework. The mass loss throughout the range of 200–800 °C was attributed to the oxidation of deposited coke (specifically, to soft carbon in the range of 200–400 °C and hard carbon in the range of 400–800 °C).^45,46^ The two catalysts evidently differed in coke content because the CZSM-5 zeolite showed a mass loss of 19.4% after removing all coke deposits, whereas the HNZSM-5 had a mass loss of 27.1%. More coke was deposited on the latter material because this specimen had more accessible acid sites, and the formation of carbonaceous species is promoted by the large number of acidic sites on the outer surfaces. Other studies have also found that mesoporous ZSM-5 zeolites form more carbon deposits than microporous ZSM-5 zeolites during catalytic reactions.^47,48^ The formation of coke results from the generation of high molecular weight compounds, and the DTG curve indicates that the HNZSM-5 produced a small peak at 290 °C. This peak may have been related to the combustion of heavy hydrocarbons aggregated on the outer surfaces of the zeolite crystals. In contrast, the peak obtained from CZSM-5 had an apex above 290 °C, presumably because some coke was located in the micropores and hence was not readily lost. The maximum mass loss of the HNZSM-5 catalyst occurred at approximately 580 °C, suggesting that the majority of the deposits comprised polycyclic aromatics produced by the polymerization/condensation reactions of aromatic hydrocarbons.^49^

## 4 Conclusions

Hydrophobic 18-carbon alkyl groups were used to synthesize a diquaternary ammonium-type surfactant (C_18–6–6_Br_2_) that was then employed as an SDA to produce HNZSM-5. This material had a larger BET surface area and better accessibility to acidic sites than CZSM-5. When applied to the catalytic cracking of a WCOMC to prepare light olefins, HNZSM-5 also exhibited improved performance and a usable reaction time of 45 h. The hierarchical pore structure and greater surface area of HNZSM-5 allowed it to outperform CZSM-5 during the catalytic cracking of macromolecules.

## Supporting Information Available

Synthesis of structure-directing agent C_18–6–6_Br_2_; TEM images of HNZSM-5 specimens produced using different crystallization times (PDF)
