# Supplementary material for: Synthesis of Two-Dimensional Zeolite Nanosheets Applied to the Catalytic Cracking of a Waste Cooking Oil Model Compound to Produce Light Olefins
Source: ACS Omega. 2024 Apr 2;9(15):17054–65. doi: 10.1021/acsomega.3c08748 (PMC11025087; doi:10.1021/acsomega.3c08748)
Supplement: Supplementary file 1 — ao3c08748_si_001.pdf [file ao3c08748_si_001.pdf]

Supporting Information

Synthesis of two-dimensional zeolite nanosheets applied to the catalytic cracking of a waste cooking oil model compound to produce light olefins

Wenbo Luo[1], Haoyu Liu[1], Hong Yuan[1,2,3] *, Hao Liu[1]

[1] School of Chemistry and Chemical Engineering, North Minzu University, Yinchuan 750021, China

[2] State Key Laboratory of National Ethnic Affairs Commission Chemical Technology, North Minzu University, Yinchuan 750021, China

[3] Ningxia Key Laboratory of Solar Chemical Conversion Technology, North Minzu University, Yinchuan, 750021, China

** Corresponding author Hong Yuan, yuanhong@nun.edu.cn*

Wenbo Luo, 444281833@qq.com

Haoyu Liu, 2507486817@qq.com

Hao Liu, 643368063@qq.com

SUPPLEMENTARY FIGURES

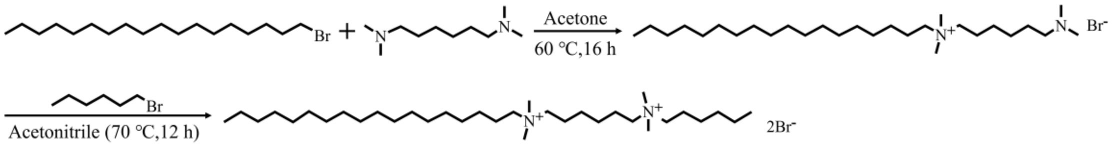

Figure S1. Synthesis of structure directing agent $C_{18-6-6}Br_2$.

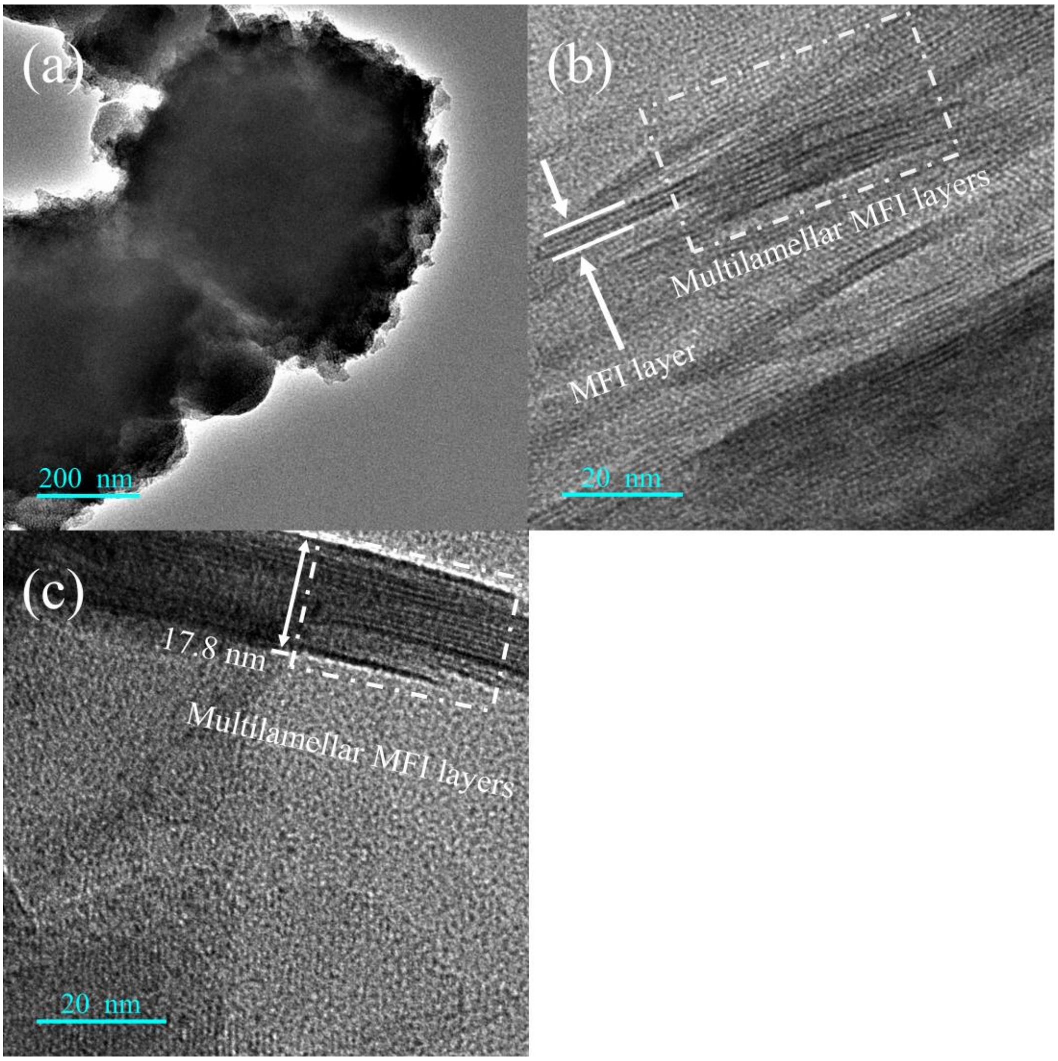

Figure S2. TEM images of HNZSM-5 specimens produced using different crystallization times. (a) 72 h; (b) and (c) 120 h.
